# Metabolomic changes associated with treatment response of neoadjuvant chemotherapy with TEC regimen in HER2-negative breast cancer

**DOI:** 10.3389/fphar.2025.1707223

**Published:** 2025-11-19

**Authors:** Kun Fang, Cuiping Wang, Zhenfeng Li, Li Wang, Xintong Wang, Zhenwei Jiang, Mengyuan Wu, Shuo Diao, Mingming Yu, Hai Yang, Sherwin K. B. Sy, Pan Deng, Qiang Mu

**Affiliations:** 1 Department of Breast surgery, Qingdao Central Hospital, University of Health and Rehabilitation Sciences, Qingdao, China; 2 School of Medicine and Pharmacy, Ocean University of China, Qingdao, China; 3 Department of Pharmaceutical Analysis, College of Pharmaceutical Sciences, Soochow University, Suzhou, Jiangsu, China; 4 Department of Pharmacy, Qingdao Central Hospital, University of Health and Rehabilitation Sciences, Qingdao, China; 5 Department of Statistics, State University of Maringá, Maringá, Paraná, Brazil

**Keywords:** TEC regimen, breast cancer, biomarker, metabolomics, treatment response

## Abstract

**Introduction:**

This study aimed to characterize time-dependent metabolic alterations and identify metabolites associated with treatment response in HER2-negative breast cancer patients undergoing neoadjuvant chemotherapy (NAC) with the TEC regimen (docetaxel, epirubicin, and cyclophosphamide).

**Methods:**

A total of 60 plasma samples were collected from 20 patients at three time points: baseline (T1), after three cycles of NAC (T2), and before surgery (T3). Pathological assessment classified patients into three response groups: pathologic complete response (pCR, n = 5), pathologic partial response (pPR, n = 7), and pathologic stable disease (pSD, n = 8).

**Results:**

After three cycles of NAC, a greater decrease in glycochenodeoxycholate was associated with poorer treatment response, whereas a larger reduction in LysoPC(18:1) correlated with better response. Following six cycles, elevated epinephrine levels were positively associated with therapeutic efficacy, while increased cysteine levels were linked to unfavorable outcomes. Ursodeoxycholic acid showed an upward trend in pCR patients but declined in pPR and pSD groups. Combined analysis of ursodeoxycholic acid and cysteine improved the predictive performance for treatment response.

**Discussion:**

These findings reveal dynamic metabolic reprogramming during NAC and suggest that ursodeoxycholic acid and cysteine may serve as potential predictive biomarkers of therapeutic efficacy in HER2-negative breast cancer patients treated with the TEC regimen.

## 1 Introduction

Neoadjuvant chemotherapy (NAC) is a preoperative treatment strategy used in breast cancer patients with axillary lymph node involvement or large tumor volumes who wish to undergo breast-conserving surgery. It aims to downstage the disease and reduce tumor size, thereby increasing the likelihood of successful surgery. A multinational survey reported that approximately 7%–27% of newly diagnosed breast cancer patients receive NAC as part of their treatment strategy (Vaidya et al., 2018). Currently, NAC has become the standard of care for patients with locally advanced breast cancer and plays a crucial role in the systemic treatment of the disease. Due to the heterogeneity of breast cancer, therapeutic responses vary across molecular subtypes and chemotherapy regimens. NAC is typically adapted from adjuvant regimens and commonly includes a combination of anthracyclines and taxanes. Among these, the TAC regimen (docetaxel, doxorubicin, and cyclophosphamide) has shown superior efficacy and is widely used as a standard adjuvant option for early-stage breast cancer (Xu et al., 2018). As a modified regimen, TEC (docetaxel, epirubicin, and cyclophosphamide) regimen replaces doxorubicin with epirubicin to reduce cardiotoxicity and has also demonstrated favorable efficacy in NAC(3–5). Approximately 20% of breast cancers are characterized by human epidermal growth factor receptor 2 (HER2) amplification or overexpression (Nader-Marta et al., 2022). For these HER2-positive patients, taxane-based chemotherapy combined with trastuzumab is the standard of care. For patients showing good clinical response, current guidelines recommend completing 4 to 6 cycles of NAC before surgery (Gradishar et al., 2024). The efficacy of NAC is primarily assessed through pathological examination of surgical specimens. Pathologic complete response (pCR) is an important indicator reflects the patient’s response to NAC and helps predict prognosis. Attaining pCR after NAC is associated with survival benefits, including prolonged overall survival, sustained disease-free survival, and significantly diminished recurrence rates (Cortazar et al., 2014).

However, the overall pCR rate among breast cancer patients remains suboptimal, ranging from 19% to 27.8%, particularly in HER2-negative (HER2-) subtypes. For instance, patients with hormone receptor-positive and HER2-negative (HR+/HER2-) breast cancer have pCR rates as low as 5%–10% (Houssami et al., 2012; Boughey et al., 2014). Therefore, HER2-breast cancer requires increased clinical and research investigation. In addition to postoperative pathology, imaging monitoring typically requires completion of 2–4 cycles of chemotherapy before tumor progression can be determined. There are no validated biomarkers for real-time assessment of treatment efficacy during therapy. As a result, drug-resistant patients may not be identified early, which delays treatment adjustments and even leads to decreased survival rates. Identifying biomarkers that can dynamically evaluate NAC efficacy, detect responsive patients, and optimize individualized treatment is essential for guiding clinical decisions.

Metabolomics offers a powerful approach to predict treatment outcomes in breast cancer. Growing evidence indicates that breast cancer development and progression are closely linked to metabolic dysregulation (Kühn et al., 2016; Wu et al., 2018). Notably, chemotherapy-induced metabolic shifts in tumors and their microenvironment often precede measurable tumor size changes (Wang et al., 2023; Shajahan-Haq et al., 2015).

Several studies have explored the potential of metabolite panels as biomarkers for early prediction of response to neoadjuvant chemotherapy in breast cancer patients. Wei et al. reported that pCR patients showed decreased threonine, glutamine, and alpha-linolenic acid, and increased isoleucine levels in serum before NAC with an anthracycline- and taxane-based sequential regimen (Wei et al., 2013). However, this study only analyzed static metabolic profiling before NAC treatment and did not consider the effects of chemotherapy on metabolic network remodeling. Díaz et al. reported that Luminal B patients who responded well to the sequential regimen showed increased lysophospholipids and decreased carnitines (Díaz et al., 2022). Glycohyocholic and glycodeoxycholic acids before surgery were identified as prognostic markers in triple-negative breast cancer (TNBC) patients, capable of effectively predicting treatment response (area under the receiver operating characteristic curve [AUC] = 0.946, 95% CI: 0.875–1) and distinguishing patients with an expected survival of more than 2 years (AUC = 0.777, 95% CI: 0.541–1). Nonetheless, the sampling time points in the study were mostly limited to before and after chemotherapy, lacking longitudinal tracking of metabolic adaptations occurring during treatment. Yamada et al. reported significantly lower baseline levels of 3-indoxyl sulfate, creatine, and urate in patients who achieved pCR (17). However, their study only vaguely described the chemotherapy regimen as “anthracycline- and taxane-based” without detailed protocol specifications (e.g., drug combinations, dosages, or cycle intervals). Furthermore, metabolic profiling was restricted to pre-treatment and the second cycle, omitting potential dynamic changes in the later stages of NAC. All these previous studies included HER2-positive patients receiving targeted therapies (e.g., trastuzumab or pertuzumab), which may confound the metabolic effects driven solely by chemotherapy and limit the interpretability of findings in HER2-populations. Therefore, metabolomics studies focusing on the TEC regimen in HER2-breast cancer patients undergoing NAC remain limited.

In this study, we employed ultrahigh performance liquid chromatography-high resolution mass spectrometry (UHPLC-HRMS) to analyze plasma metabolomic changes at three time points: before neoadjuvant chemotherapy (NAC), after the third cycle, and prior to surgery in patients with HER2-breast cancer receiving the TEC regimen. We further examined the correlation between these metabolic changes and treatment efficacy.

## 2 Methods

### 2.1 Study cohort and design

The study was conducted in Qingdao Central Hospital between December 2023 and June 2024. 20 patients with primary breast cancer were recruited to participate. Inclusion criteria were as follows: (Vaidya et al., 2018) female patients aged ≥18 years (Xu et al., 2018); histologically or cytologically confirmed primary locally advanced breast cancer, defined according to the American Joint Committee on Cancer (AJCC) TNM staging system as T2–T4 (tumor size >2 cm), N0–N3 (lymph node status ranging from no involvement to extensive regional nodal involvement, and M0 (no distant metastasis) (Gu X. et al., 2015); HER2-negative status was confirmed by immunohistochemistry (IHC 0 or 1+ or IHC 2+ with negative *in situ* hybridization) (Liu et al., 2019); no prior treatment for breast cancer, except diagnostic biopsy or surgery for benign breast disease (Li B. et al., 2021); scheduled to receive the TEC regimen. Exclusion criteria included (Vaidya et al., 2018): stage IV (metastatic) breast cancer (Xu et al., 2018); inflammatory breast cancer (Gu X. et al., 2015); bilateral breast cancer (Liu et al., 2019); history of other malignancies within the past 2 years.

All patients received 6 cycles of NAC with a regimen of epirubicin 90 mg/m^2^, cyclophosphamide 600 mg/m^2^ and docetaxel 80 mg/m^2^ intravenously every 3 weeks. Surgery was performed within 21 days after completion of NAC. The surgical procedure was a modified radical surgery, with breast-conserving surgery as an option for patients with breast-conserving conditions.

Clinical information of the enrolled patients was collected, including age, gender, presence of metabolic diseases, histologic grading, hormone receptor expression, HER2 status, Ki-67 expression, tumor node metastasis (TNM) information, and imaging results (ultrasound, mammography, magnetic resonance imaging, and computed tomography). Information on Miller-Payne (MP) grading, Residual Cancer Burden (RCB) grading and post-neoadjuvant pathological TNM staging (ypTNM) was collected from patients’ postoperative pathology reports.

The study was conducted in accordance with the Declaration of Helsinki, International Conference on Harmonization and Good Clinical Practice guidelines, and approved by the Medical Ethics Committee of Qingdao Central Hospital (KY202321002), and all patients signed a written informed consent prior to any protocol-related procedures and treatments.

### 2.2 NAC response evaluation

Patients underwent breast ultrasonography before each treatment cycle and mammography every two cycles during NAC. Treatment response was evaluated through radiographic tumor size changes, with tumor burden quantified by the sum of the longest diameter of target lesions according to the RECIST 1.1 criteria. A comparative analysis was performed between pretreatment core needle biopsy specimens and surgically resected tumors. Following the completion of neoadjuvant therapy, histopathological response was evaluated using the Miller-Payne (MP) grading system and residual cancer burden (RCB) classification. pCR was defined as the absence of invasive carcinoma in the primary tumor bed (ductal carcinoma *in situ* permitted) with negative axillary lymph nodes, corresponding to MP grade 5 with nodal clearance or RCB class 0. Pathological partial response (pPR) was defined as MP grades 3–4 with RCB class I-II, and pathological stable disease (pSD) was defined as MP grades 1–2 with RCB class III.

### 2.3 Sample collection

A total of 60 plasma samples were collected from 20 patients at three time points during NAC (baseline, T1; after 3 cycles, T2; and after 6 cycles, before surgery, T3). Patients fasted after 10 p.m. the night before sample collection. On the following morning, 2 mL of fasting venous blood sample was collected in an EDTA anticoagulant tube for plasma separation. After centrifugation at 1,600 *g* for 10 min, the upper plasma layer was separated and divided into three aliquots of no less than 0.2 mL each. All samples were stored at −80 °C until further analysis.

### 2.4 Metabolite extraction and UHPLC-HRMS analysis

After slowly thawing the samples at 4 °C, 100 µL of plasma sample was added to 400 µL of pre-cooled methanol/acetonitrile/water mixture (2:2:1, v/v) (Zhang et al., 2021; Li R. et al., 2021). The mixture was vortexed and subjected to low-temperature ultrasound for 30 min, followed by a 10-min incubation at −20 °C. The samples were then centrifuged at 14,000 × g at 4 °C for 20 min. The supernatant was vacuum-dried, and for mass spectrometry analysis, 100 μL of acetonitrile-water solution (acetonitrile: water = 1:1, v/v) was added for reconstitution. The mixture was vortexed, centrifuged at 14,000×g at 4 °C for 15 min, and the supernatant was collected for analysis.

After separation with the Vanquish LC ultrahigh performance liquid chromatography (UHPLC) system, using a Waters ACQUITY UPLC BEH Amide column (1.7 μm, 2.1 × 100 mm), mass spectrometry analysis was performed using the Orbitrap Exploris™ 480 mass spectrometer (Thermo Scientific, CA, United States). Both positive and negative ion modes were used for electrospray ionization (ESI) detection. The chromatography conditions were as follows: column temperature 25 °C; flow rate 0.5 mL/min; injection volume 2 μL; mobile phase A: water containing 25 mM ammonium acetate and 25 mM ammonia hydroxide; mobile phase B: acetonitrile. The gradient elution program was as follows: 0–0.5 min, 95% B; 0.5–7 min, B linearly decreased from 95% to 65%; 7–8 min, B linearly decreased from 65% to 40%; 8–9 min, B maintained at 40%; 9–9.1 min, B linearly increased from 40% to 95%; 9.1–12 min, B maintained at 95%. During the entire analysis, the samples were kept in the 4 °C autosampler. To minimize the effect of instrument signal fluctuations, the samples were analyzed in a random order. QC samples were inserted into the sample queue to monitor and evaluate the system’s stability and the reliability of experimental data. For QC, 10 µL of supernatant from each plasma extract was pooled to generate the QC sample, with a total of eight QC samples prepared and injected after every ten study samples throughout the analytical sequence.

The ESI source and mass spectrometry parameters were set as follows: sheath gas, 50; auxiliary gas, 2; ion transfer tube temperature, 350 °C; spray voltage, 3500 V in positive ion mode and 2800 V in negative ion mode. The m/z range for primary mass-to-charge ratio detection was 70–1,200 Da, with a resolution of 60,000 and a scan accumulation time of 100 m. MS/MS acquisition was performed using a stepped scan approach with a scan range of 70–1,200 Da, with a resolution of 60,000. The scan accumulation time was 100 m, and the dynamic exclusion time was set to 4 s.

### 2.5 Data processing and analysis

The raw data were converted into mzXML format using ProteoWizard (version 3.0), followed by peak alignment, retention time correction, and peak area extraction using the XCMS Online (version 3.7.1). The data extracted by XCMS were first used for metabolite identification and data preprocessing (removing ion peaks with missing values >50%; imputing missing values using k-nearest neighbors (KNN); filtering features with RSD >50%). Following this, the quality of the experimental data was evaluated, and then data analysis was conducted.

One-way ANOVA was employed to compare normally distributed continuous variables across multiple groups, while the Chi-square test or Fisher’s exact test was applied to compare categorical variables in other clinicopathological features. The significance of metabolites in two groups was calculated by T-test. Data were normalized and subjected to principal component analysis (PCA) using the MetaboAnalyst 6.0 platform to visualize clustering relationships among all samples. Partial least squares discriminant analysis (PLS-DA) was used to differentiate the metabolic features among three groups of samples. One sample from the T1 group was located outside the 95% Hotelling’s T^2^ confidence interval in the PLS-DA score plot. However, upon further inspection of raw data quality indicators, including total peak counts and clustering behavior with QC samples, no technical artifacts or abnormal signals were detected. In line with the QComics guidelines, which advocate retaining biologically plausible variability while minimizing artificial bias, this sample was retained to preserve dataset integrity and avoid unjustified exclusion (González-Domínguez et al., 2024). In the PLS-DA model, metabolites with a variable importance in projection (VIP) value greater than 1.0 (VIP >1.0) in the first principal component were considered as potential differential metabolites. The selection of significantly differential metabolites between groups was based on the thresholds of fold change (FC > 1.2 or FC < 0.833) and *p* < 0.05, as referenced from a recent metabolomics study using human plasma samples (An et al., 2022). This criterion, commonly applied in metabolomics, allows the detection of subtle but consistent biological differences and improves sensitivity, particularly when the sample size is limited. KEGG pathway enrichment analysis was conducted on the differentially expressed metabolites. Butterfly plots were generated using the bioinformatics cloud platform tools (Applied Protein Technology, Shanghai, China) to visualize the distribution patterns of upregulated and downregulated metabolites within enriched metabolic pathways. The predictive performance of individual metabolites and the combined model was evaluated by receiver operating characteristic (ROC) curve analysis using R script (version 4.3.1) packages including pROC, ggplot2 and glmnet.

## 3 Results

### 3.1 Patient clinical characteristics

A total of 20 patients at initial diagnosis of HER2-breast cancer was enrolled, with a median age of 46 (range: 29–60) years. Six patients were aged ≥50 years. There were 6 TNBC patients and 14 HR+/HER2-patients. Postoperative pathological evaluation following completion of NAC and surgery showed that 5 patients achieved pCR, 7 achieved pPR, and 8 achieved pSD. The pCR rate for TNBC patients was 50%, while the pCR rate for HR+/HER2-patients was 14.29%. The detailed demographic and clinicopathological characteristics of the participants are listed in Table 1. Except for the pSD group, which had a significantly higher clinical stage than the pCR group (p = 0.034), no significant differences were observed in other clinical characteristics among the efficacy groups.

**TABLE 1:** Clinicopathological characteristics of the study population.

Characteristics	All (n = 20) number (%)	pCR (n = 5) number (%)	pPR (n = 7) number (%)	pSD (n = 8) number (%)	P value^a^
Age at diagnosis (median, range)	46 (29–60)	46 (33–54)	48 (40–60)	42 (29–57)	0.472^b^
Menopausal status					1.000^c^
Pre-	14 (70.0)	4 (80.0)	5 (71.4)	5 (62.5)	
Post-	6 (30.0)	1 (20.0)	2 (28.6)	3 (37.5)	
Subtype					0.232^c^
Triple-negative	6 (30.0)	3 (60.0)	2 (28.6)	1 (12.5)	
HR+/HER2-	14 (70.0)	2 (40.0)	5 (71.4)	7 (87.5)	
Clinical T stage					0.299^d^
cT2	15 (75.0)	5 (100.0)	5 (71.4)	5 (62.5)	
cT3	3 (15.0)	0 (0)	2 (28.6)	1 (12.5)	
cT4	2 (10.0)	0 (0)	0 (0)	2 (25.0)	
Clinical nodal stage					0.037^d^
cN0	6 (30.0)	3 (60.0)	3 (42.9)	0 (0)	
cN1	13 (65.0)	2 (40.0)	4 (57.1)	7 (87.5)	
cN2	1 (5.0)	0 (0)	0 (0)	1 (12.5)	
Metabolic disease					0.387^c^
Yes	4 (20.0)	0 (0)	1 (14.3)	3 (37.5)	
No	16 (80.0)	5 (100.0)	6 (85.7)	5 (62.5)	
Ki-67					0.189^c^
≤20%	6 (30.0)	0 (0)	2 (28.6)	4 (50.0)	
>20%	14 (70.0)	5 (100.0)	5 (71.4)	4 (50.0)	
Stage					0.025^d^
IIA	5 (25.0)	3 (60.0)	2 (28.6)	0 (0)	
IIB	11 (55.0)	2 (40.0)	4 (57.1)	5 (62.5)	0.034^e^
IIIA	2 (10.0)	0 (0)	1 (14.3)	1 (12.5)	
IIIB	2 (10.0)	0 (0)	0 (0)	2 (25.0)	
Histological grade					0.298^c^
G2	13 (65.0)	2 (40.0)	6 (85.7)	5 (62.5)	
G3	7 (35.0)	3 (60.0)	1 (14.3)	3 (37.5)	

^a^ P values from overall comparisons (pCR, vs. pPR, vs. pSD).

^b^ One-way ANOVA.

^c^ Fisher-Freeman-Halton exact test.

^d^ Kruskal–Wallis H test.

^e^ Post hoc pairwise comparison (pSD, vs. pCR): Dunn’s test with Bonferroni adjustment.

### 3.2 Analysis of imaging changes in tumor size during NAC

**FIGURE 1:**
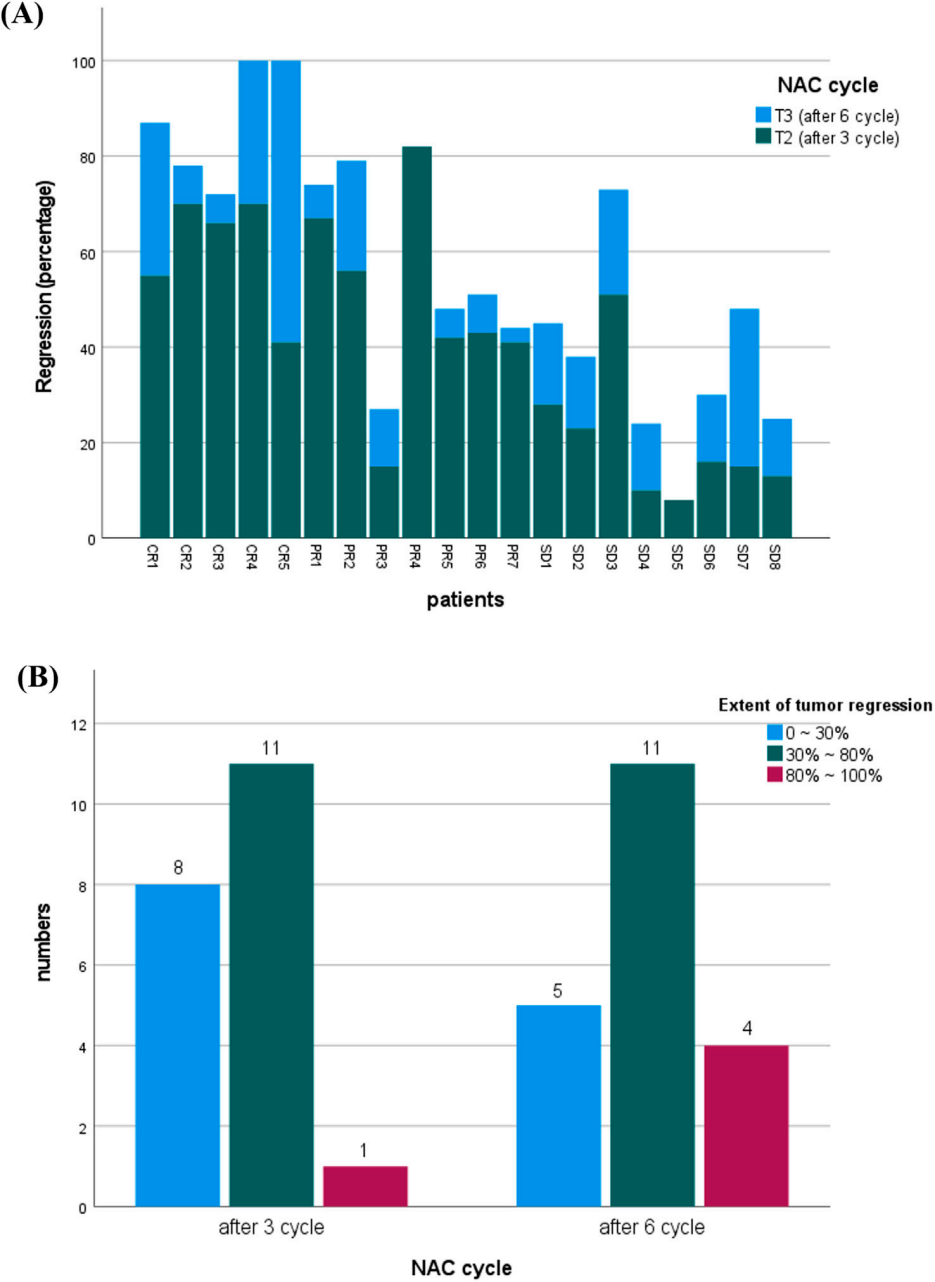
**(A)** Comparison of tumor regression percentages in patients after three versus six cycles of neoadjuvant chemotherapy (NAC). **(B)** Distribution of patients by tumor regression extent (0%–30%, 30%–80%, 80%–100%) following three and six cycles of NAC. Tumor size and regression data were assessed via ultrasound imaging.

No significant differences in tumor size were observed between the groups at baseline (Table 2). However, after three and six cycles of NAC, the pCR group exhibited significantly smaller tumors compared to the pPR and pSD groups, indicating that pCR had the best therapeutic response. Tumor size progressively decreased throughout NAC treatment. Moreover, the degree of tumor regression from baseline was significantly greater in the pCR group after three and six cycles, demonstrating the highest reduction among all groups (Figure 1A). As shown in Figure 1B, tumor regression after three cycles of NAC varied widely, with most patients (n = 11) achieving 30%–80% regression, while a smaller subset (n = 8) showed minimal regression (0%–30%). Only one patient reached near-complete regression (80%–100%). After six cycles, the response improved: more patients (n = 4) achieved 80%–100% remission, and fewer (n = 5) remained in the low-response group (0%–30%).

**TABLE 2:** Imaging changes in tumor size during NAC.

Parameter	All	pCR	pPR	pSD	P value^a^
T1 (mm)	37.50 ± 18.95	25.20 ± 7.92	37.43 ± 10.16	45.25 ± 25.96	0.141
T2 (mm)	22.60 ± 16.93	10.40 ± 5.59	17.14 ± 4.10	35.00 ± 20.84	0.006
T3 (mm)	16.00 ± 12.22	3.00 ± 2.74	14.29 ± 4.68	25.63 ± 12.36	0.001
T2 vs. T1 regression (percentage)	40.60% ± 23.50%	60.40% ± 12.46%	49.43% ± 21.47%	20.50% ± 13.97%	0.012
T3 vs. T1 regression (percentage)	56.65% ± 27.08%	87.40% ± 12.68%	57.86% ± 20.73%	36.38% ± 19.57%	0.004

^a^ Dunn’s *post hoc* test (Bonferroni-adjusted) for pSD, vs. pCR, following a significant Kruskal–Wallis H test.

### 3.3 Longitudinal screening of differential metabolites across time in NAC

**FIGURE 2:**
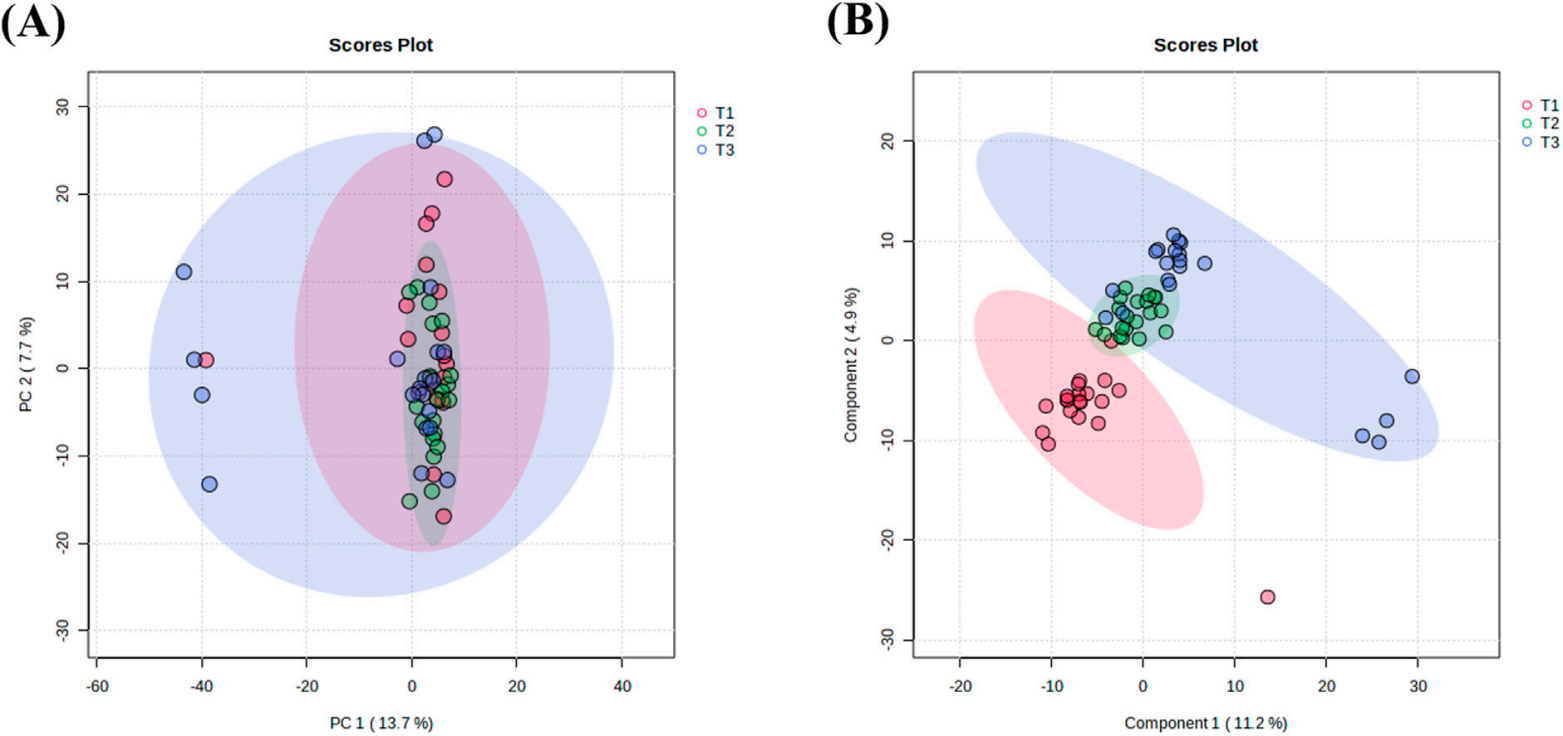
Multivariate analysis of NAC treatment timepoints. **(A)** PCA score plot showing sample clustering among different NAC treatment cycles. **(B)** PLS-DA score plot illustrating inter-group separations across chemotherapy phases. The elliptical areas represent 95% confidence regions, with colored points indicating sample distributions of corresponding groups. T1: pre-NAC samples (pink); T2: post-3-cycle NAC specimens (green); T3: pre-operative samples after 6 NAC cycles (blue).

UHPLC-HRMS analysis identified 1,147 metabolites in plasma samples. Principal component analysis (PCA) was conducted on 60 plasma samples from 20 breast cancer patients to assess metabolic changes across three NAC time points (T1-T3). The PCA score plots showed a relatively overlapping distribution of samples, which did not clearly distinguish between the groups (Figure 2A). Permutational multivariate analysis of variance indicated statistically significant differences in overall metabolic profiles across different NAC treatment time points (F = 2.8842, p = 0.029), despite substantial inter-individual metabolic variability (*R* ^2^ = 0.09). Supervised PLS-DA was performed to further characterize metabolic changes (Figure 2B). The results revealed clear separation between pre-NAC (T1, red) and post-NAC (T3, blue) samples, confirming significant NAC-induced metabolic alterations. Mid-NAC samples (T2, green) exhibited an intermediate distribution, with partial overlap between pre- and post-NAC clusters, reflecting progressive metabolic remodeling during NAC. Notably, a subset of post-NAC samples maintained transitional features, indicating interpatient variability in response to extended chemotherapy.

**FIGURE 3:**
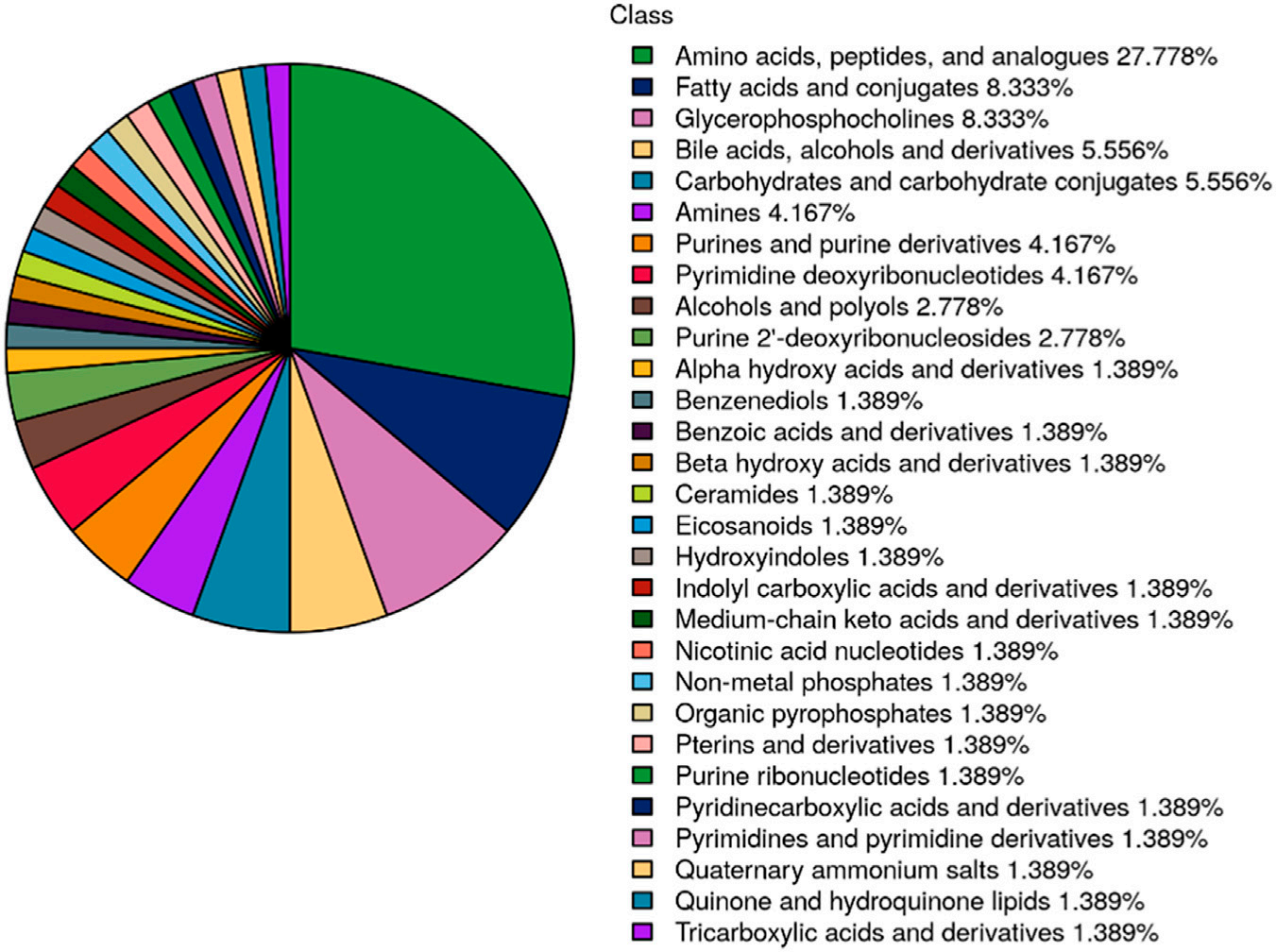
Pie chart displaying the distribution of differentially metabolites across 3 NAC time points (T1: pre-NAC; T2: after 3 cycles; T3: after 6 cycles, before surgery). Metabolites are categorized by chemical class, with percentage representation of each category reflecting its proportional in the total differentially metabolites.

Seventy-five potential differential metabolites were preliminarily identified across the three NAC time points based on VIP values (>1) from component 1 of the PLS-DA model. As illustrated in Figure 3, these metabolites were categorized by chemical class, with amino acids representing the most abundant group, followed by fatty acids. Glycerophosphorylcholine and bile acids also constituted significant proportions, potentially reflecting NAC-specific metabolic alterations.

**FIGURE 4:**
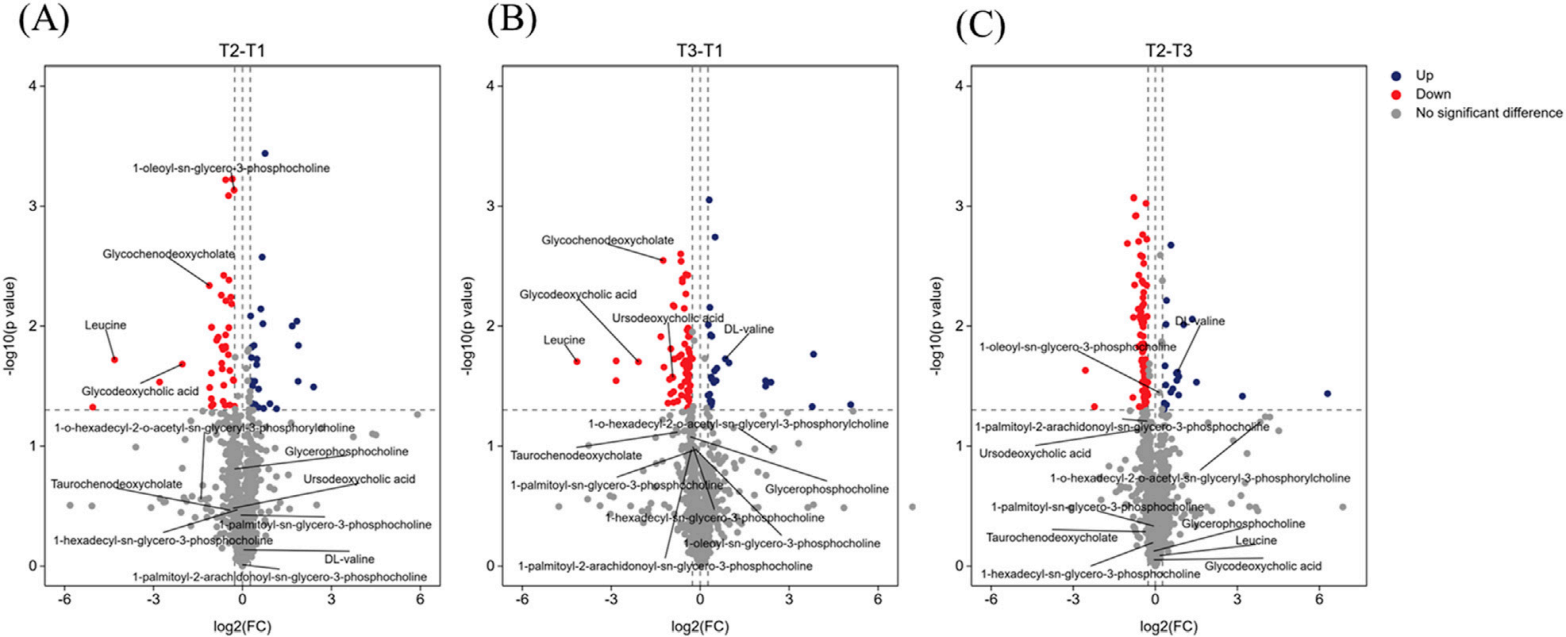
Volcano plot showing several labeled differential metabolites. **(A)** Volcano plot comparing baseline (T1) with mid-NAC (T2) phase. **(B)** Volcano plot comparing baseline (T1) with post-NAC (T3) phase. **(C)** Volcano plot comparing mid-NAC (T2) phase with post-NAC (T3) phase. The volcano plot shows log2 (fold change) on the x-axis and -log10 (p value) on the y-axis. Dashed thresholds indicate significance boundaries (|log2FC| > 0.263, p < 0.05). Red indicates significantly upregulated metabolites, blue indicates significantly downregulated metabolites, and gray did not achieve significant change. Annotated metabolite classes: glycerophosphorylcholines; bile acids; branched-chain amino acids.

Figure 4 illustrates metabolic alterations in volcano plots, comparing baseline with mid-NAC (T2-T1, Figure 4A), baseline with post-NAC (T3-T1, Figure 4B), and mid-NAC with post-NAC (T2-T3, Figure 4C) time points. Among branched-chain amino acids, leucine demonstrated significant reduction throughout NAC treatment. Valine showed a decreasing trend at mid-treatment (T2) but rebounded to significantly elevated levels post-treatment (T3), while glycerophosphorylcholine levels tended to decrease post-NAC compared to baseline, these changes did not reach statistical significance. In contrast, bile acids exhibited significant downregulation following NAC treatment.

### 3.4 Dynamic changes in plasma metabolites and related pathways enrichment analysis

**FIGURE 5:**
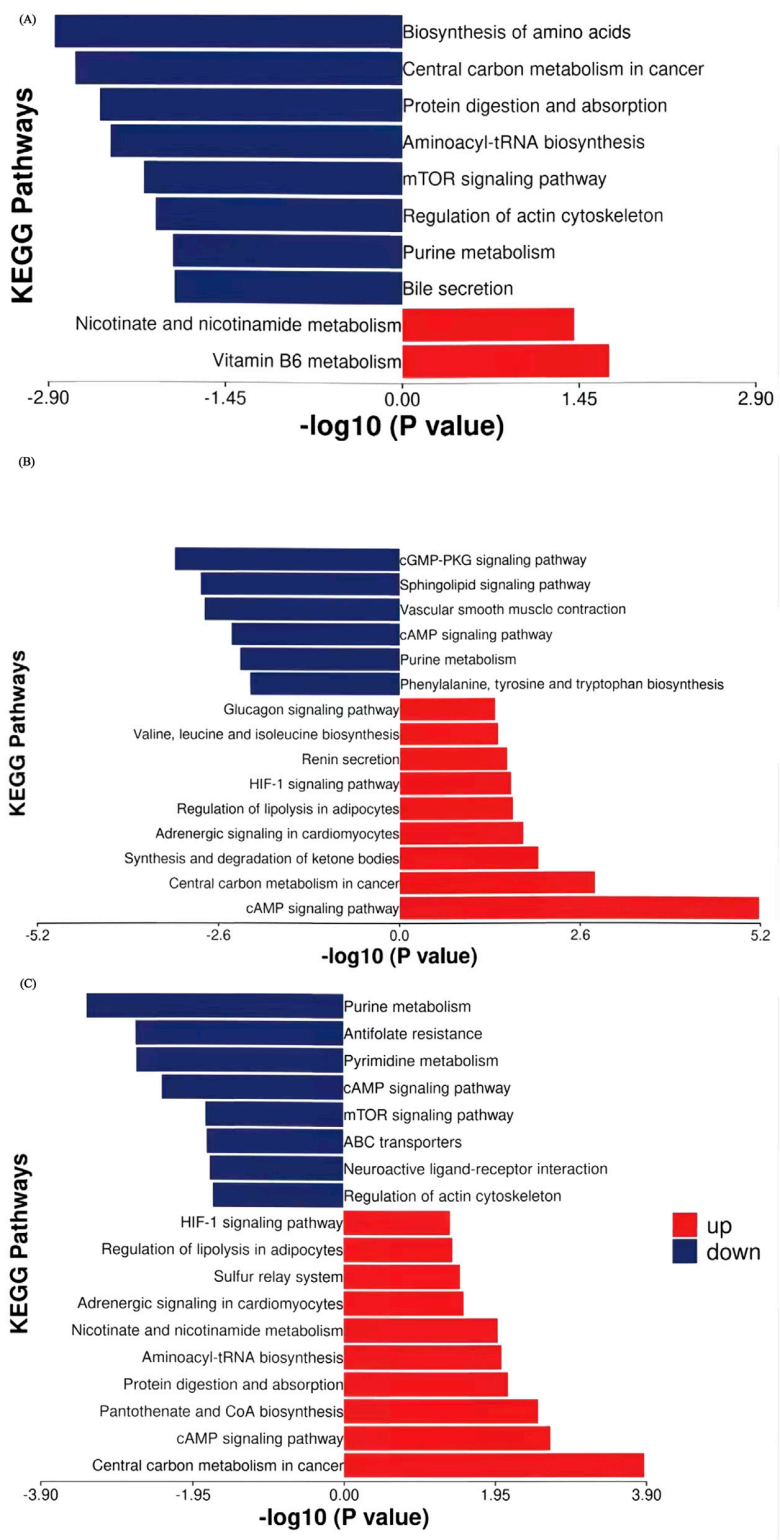
KEGG pathway enrichment analysis of stage-specific differential metabolites with thresholds of p < 0.05 and |log2FC| > 0.263. **(A)** First 3 cycles of NAC (T2 vs. T1). **(B)** Cycles 4-6 of NAC (T3 vs. T2). **(C)** Full NAC course (T3 vs. T1). Y-axis: Metabolic pathways ordered by enrichment significance. X-axis: Enrichment significance (-log10 (p-value)). Red and blue: Pathways enriched with upregulated and downregulated metabolites, respectively.

To characterize metabolic dynamic changes during NAC, we conducted paired t-tests on the 75 potential differential metabolites identified via PLS-DA, using T1 as the baseline for longitudinal comparison at T2 and T3. Metabolites meeting criteria of fold change >1.2 (or <0.833) with p < 0.05 were classified as significantly altered. KEGG pathway enrichment analysis revealed the biological significance of up- and downregulated metabolites between time points (Figure 5). Eleven metabolites showed significant changes at T2 versus T1, increasing to thirty metabolites at T3 versus T1. Seven metabolites showed persistent alterations across both treatment phases, with changes initiating in T2 and persisting until T3. Those downregulated metabolites included leucine, inosine 5′-diphosphate, glycochenodeoxycholate, glycodeoxychycholic acid, and acetylcholine, while the upregulated metabolites were nicotinate D-ribonucleotide and N-acetyl-glucosamine 6-phosphate. In addition, 25 metabolites demonstrated late-phase NAC-specific changes (T2-T3 comparison), suggesting continued metabolic reprogramming in response to NAC throughout treatment.

During the first three NAC cycles (T2 vs. T1, Table 3), leucine (FC = 0.050, p = 0.019), glutamine (FC = 0.728, p = 0.004), inosine 5′-diphosphate (FC = 0.722, p = 0.001), and glycochenodeoxycholate (FC = 0.461, p = 0.005) showed significant downregulation. Conversely, nicotinate D-ribonucleotide (FC = 3.669, p = 0.029) and 4-pyridoxic acid (FC = 3.695, p = 0.014) showed marked upregulation. Pathway enrichment analysis identified amino acid biosynthesis and central carbon metabolism in cancer are the top two significantly affected pathways (Figure 5A). Other enriched pathways included the purine metabolism and mTOR signaling pathway, which regulates cell metabolism, proliferation, growth, apoptosis, and autophagyBile secretion was also significantly involved. The upregulated metabolites enriched belonged to pathways of nicotinate and nicotinamide metabolism as well as vitamin B6 metabolism.

**TABLE 3:** Significantly altered metabolites in plasma from patients in the first three cycles of NAC (T2 vs. T1).

Category	Metabolite name	FC value	P value	VIP
Amino acids and derivatives	Leucine	0.050	0.019	2.486
	O-acetyl-serine	0.776	0.007	1.591
	Glutamine	0.728	0.004	1.445
Nucleotide metabolism	Nicotinate d-ribonucleotide	3.669	0.029	2.542
	Inosine 5′-diphosphate	0.722	0.001	1.998
Bile acids	Glycochenodeoxycholate	0.461	0.005	2.937
	Glycodeoxycholic acid	0.245	0.021	2.573
	Acetylcholine	0.813	0.028	2.765
Glycerophosphocholines	1-Oleoyl-sn-glycero-3-phosphocholine (LysoPC(18:1))	0.821	0.001	1.297
Vitamins and cofactors	4-Pyridoxic acid	3.695	0.014	1.520
Carbohydrate metabolism	N-Acetyl-glucosamine 6-phosphate	1.234	0.018	2.141

Significantly different metabolites in the 4-6 cycles of NAC (T3 vs. T2, Table 4; Figure 5B) revealed key shifts in energy metabolism, amino acid/nucleic acid processing, and signaling pathway activity. For example, phosphoribosyl pyrophosphate (FC = 0.575, p = 0.008), deoxyinosine (FC = 0.723, p = 0.003), tryptophan (FC = 0.754, p = 0.014), myo-inositol 1,4,5-trisphosphate (FC = 0.773, p = 0.036) and adenosine (FC = 0.649, p = 0.007) showed significant downregulation. These metabolites were enriched in purine metabolism, phenylalanine, tyrosine and tryptophan biosynthesis, sphingolipid signaling and cGMP-PKG signaling pathways, implying that NAC may hinder tumor growth by disrupting nucleic acid metabolism and cell signaling. In contrast, lactate (FC = 1.482, p = 0.036), 3-hydroxybutyric acid (FC = 1.814, p = 0.026), and valine (FC = 1.758, p = 0.024) were significantly upregulated. These metabolites were enriched in pathways such as cAMP signaling, central carbon metabolism in cancer, glucagon signaling, ketone body synthesis and degradation, regulation of lipolysis in adipocytes, and branched-chain amino acid metabolism, suggesting that tumors may adapt to energy demands by modulating these pathways during the later 3 cycles of NAC. Additionally, stress hormone pathways (epinephrine/renin systems) showed enrichment, with upregulation of epinephrine (FC = 1.304, p = 0.031), indicating that NAC may influence systemic metabolic responses through stress hormone regulation.

**TABLE 4:** Significantly altered metabolites in plasma from patients in cycles 4-6 of NAC (T3 vs. T2).

Category	Metabolite name	FC value	P value	VIP
Amino acids and derivatives	4-Hydroxy-proline	0.778	0.019	2.369
	Valine	1.758	0.024	2.357
	Gamma-glutamylcysteine	0.745	0.005	1.744
	Tryptophan	0.754	0.014	1.508
	N6-methyl-lysine	0.748	0.009	1.469
	Phe-phe	78.689	0.037	1.977
Nucleotide metabolism	5-methyl-5,6-dihydrouracil	0.762	0.027	2.301
	2′-deoxycytidine 5′-monophosphate	0.579	0.001	1.824
	Phosphoribosyl pyrophosphate	0.575	0.008	1.764
	Purine	0.806	0.002	1.645
	Adenosine	0.649	0.007	1.568
	7-methylguanine	0.774	0.023	1.476
	Deoxyinosine	0.723	0.003	1.237
Carbohydrate and energy metabolism	Glucarate	0.675	0.008	2.219
	Glucosamine 6-phosphate	0.736	0.012	1.619
	Lactic acid	1.482	0.036	1.386
	Myo-inositol 1,4,5-trisphosphate	0.773	0.036	1.998
	2-keto-D-Gluconic acid	1.255	0.044	1.967
Sphingolipids	Phytosphingosine	0.493	0.002	1.306
	N-palmitoyl-sphingosine	0.696	0.008	1.576
Redox cofactors	Coenzyme q2	0.726	0.002	2.450
	Tetrahydro-biopterin	1.328	0.006	1.110
Organic amines	Triethanolamine	1.806	0.038	1.754
Ketone	3-Hydroxybutyric acid	1.814	0.026	1.426
Neurotransmitters	Epinephrine	1.304	0.031	1.471

Throughout the complete NAC regimen (T3 vs. T1, Table 5; Figure 5C), purine and pyrimidine metabolism pathways demonstrated the most pronounced suppression among downregulated pathways. This persistent suppression, consistent with patterns observed during initial treatment cycles, indicating sustained disruption of nucleic acid synthesis as a mechanism of tumor growth control. Key metabolites including thymidine 5′-monophosphate (TMP, FC = 0.743, p = 0.034), phosphoribosyl pyrophosphate (PRPP, FC = 0.716, p = 0.025), inosine 5′-diphosphate (IDP, FC = 0.793, p = 0.031) and adenosine (FC = 0.702, p = 0.029) showed significant depletion. In contrast, upregulated pathways reflected metabolic reprogramming toward energy production and biosynthetic processes. Elevated lactate levels (FC = 1.449, p = 0.029) indicated enhanced glycolysis flux. Additionally, aminoacyl-tRNA synthesis pathways, exemplified by valine (FC = 1.800, p = 0.019), and adipocyte lipolysis were activated, while protein digestion and absorption pathways showed increased activity, suggesting enhanced protein turnover. Nicotinate D-ribonucleotide (FC = 5.226, p = 0.029) showed dramatic elevation in nicotinamide metabolism, while elevated cysteine (FC = 1.234, p = 0.001) was associated with pantothenate/CoA biosynthesis pathways, implying enhanced NAD^+^ metabolism and coenzyme synthesis post-chemotherapy.

**TABLE 5:** Significantly altered metabolites in plasma from patients across the full six cycles of NAC (T3 vs. T1).

Category	Metabolite name	FC value	P value	VIP
Bile acids	Glycochenodeoxycholate	0.421	0.003	2.937
	Glycodeoxycholic acid	0.236	0.020	2.573
	Ursodeoxycholic acid	0.527	0.027	1.574
Amino acids and derivatives	Cysteine	1.234	0.001	2.830
	Leucine	0.056	0.02	2.486
	Valine	1.800	0.019	2.357
	N-methyl-alanine	1.254	0.007	2.161
	Phe-phe	33.741	0.045	1.977
	γ-glutamylcysteine	0.773	0.021	1.744
	4-Hydroxy-proline	0.716	0.004	2.369
	1-methyl-histidine	1.312	0.012	1.583
Nucleotide metabolism	Nicotinate d-ribonucleotide	5.226	0.029	2.542
	Inosine 5′-diphosphate	0.793	0.031	1.998
	2′-deoxycytidine 5′-monophosphate	0.634	0.003	1.824
	Thymidine 5′-monophosphate	0.743	0.034	1.814
	Phosphoribosyl pyrophosphate	0.716	0.025	1.764
	Adenosine	0.702	0.029	1.568
	Deoxyinosine	0.776	0.035	1.237
	5-methyl-5,6-dihydrouracil	0.736	0.011	2.301
Carbohydrate and energy metabolism	2,3-Dehydro-2-deoxy-N-acetylneuraminic acid	14.145	0.017	1.871
	Glucarate	0.705	0.019	2.219
	N-Acetyl-glucosamine 6-phosphate	1.399	0.024	2.141
Organic acids	2-Keto-gluconic acid	1.258	0.042	1.967
	Lactic acid	1.449	0.029	1.386
Fatty acids	Heptadecanoic acid	1.274	0.012	2.055
Sphingolipids	Phytosphingosine	0.591	0.042	1.306
Coenzymes	Coenzyme q2	0.715	0.005	2.450
Organic amines	Triethanolamine	1.960	0.020	1.754
Neurotransmitters	Epinephrine	1.306	0.044	1.471
	Acetylcholine	0.747	0.004	2.765

### 3.5 Metabolite changes associated with treatment response

**FIGURE 6:**
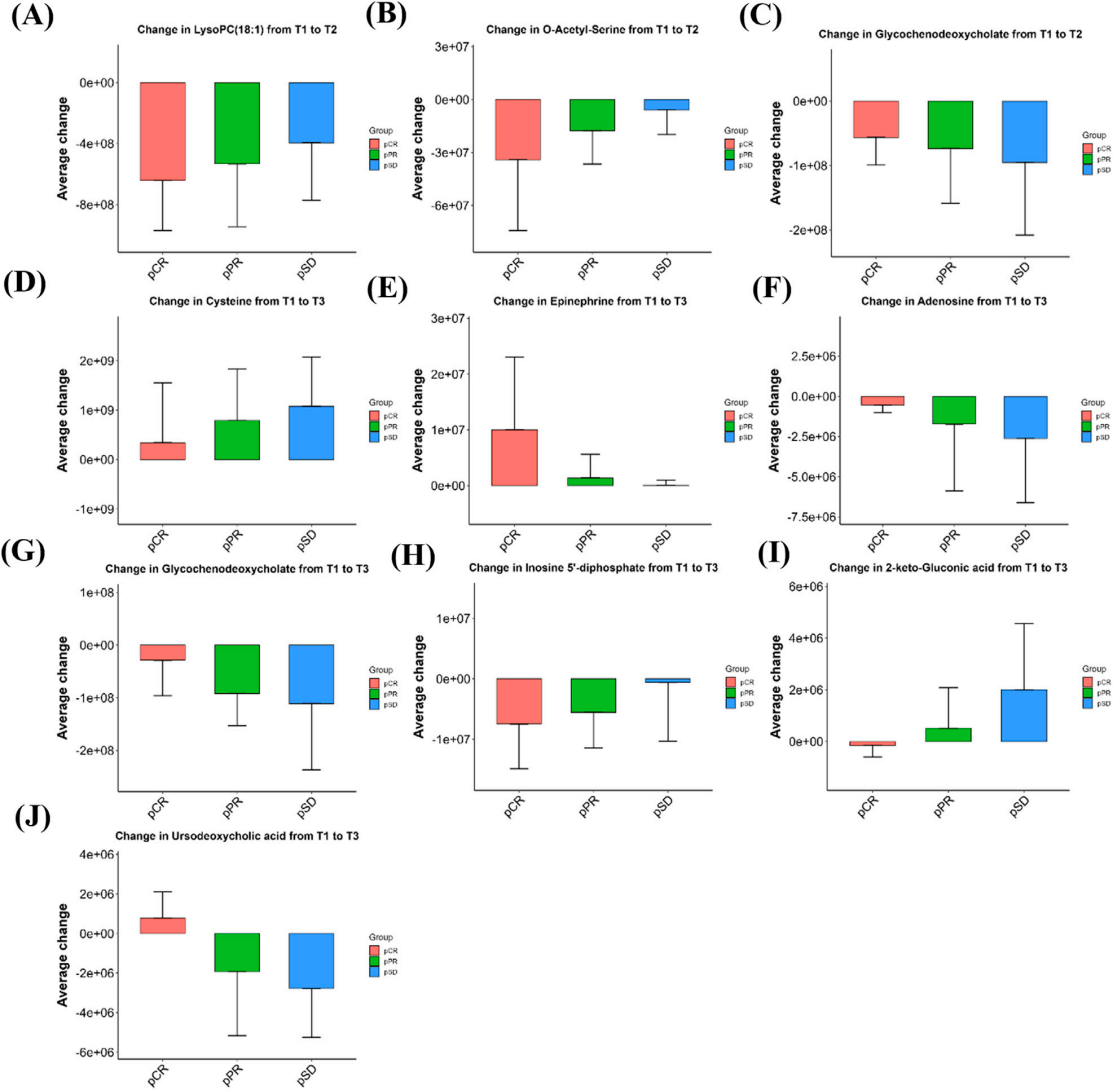
Bar graphs showing the magnitude of metabolite changes at T2 **(A–C)** and T3 **(D–J)** time points compared to baseline (T1) in the three efficacy groups of patients. Positive values indicate upregulation, negative values indicate downregulation, bar heights represent within-group sample means, and error bars are means ±1 standard deviation. pCR: pathological complete remission; pPR: pathological partial remission; pSD: pathological stable disease; T1: baseline; T2: after 3 cycles; T3: after 6 cycles.

For metabolites that exhibited significant longitudinal changes from baseline (T1) to post-treatment time points (T2 or T3), we further examined their average change across different response groups (pCR, pPR, pSD). As shown in Figure 6, these selected metabolites displayed increasing or decreasing trends in mean change values from pCR to pSD. Although the differences between groups were not statistically significant, these gradient patterns may indicate potential metabolic signatures associated with treatment response. After three cycles of NAC (T2), three metabolites showed decreasing levels. O-acetyl-serine and 1-oleoyl-sn-glycero-3-phosphocholine (LysoPC(18:1)) observed the largest reductions in the pCR group (strongest responders), followed by pPR and the smallest changes in pSD. In contrast, glycochenodeoxycholate exhibited an inverse pattern, with the most pronounced reduction in pSD (weakest responders), moderate reduction in pPR, and minimal change in pCR.

As illustrated in Figures 6D–J, cysteine exhibited a progressively greater increase from the pCR group to the pSD group after six cycles of NAC (T3). Epinephrine was also elevated across all groups, with the highest mean change in the pCR group and the lowest in pSD. Conversely, adenosine and glycochenodeoxycholate showed reduced levels post-treatment, with larger average decreases in pSD compared to pPR and pCR. Inosine 5′-diphosphate (IDP) also showed a downward trend, with the extent of decline appearing greater in pCR than in pPR or pSD. Additionally, 2-keto-gluconic acid decreased in pCR patients but increased in pPR and pSD groups, while ursodeoxycholic acid showed an opposite trend, increasing in pCR and decreasing in non-pCR patients. The relative intensity changes of these metabolites across the three time points (T1, T2, T3) for each efficacy group are shown in Supplementary Figure S1. The mean relative abundance of all 1,147 detected metabolites in each group, together with the fold changes between groups and the corresponding P values, are presented in the Supplementary Table S1.

### 3.6 ROC analysis and joint prediction of efficacy-related metabolites

**FIGURE 7:**
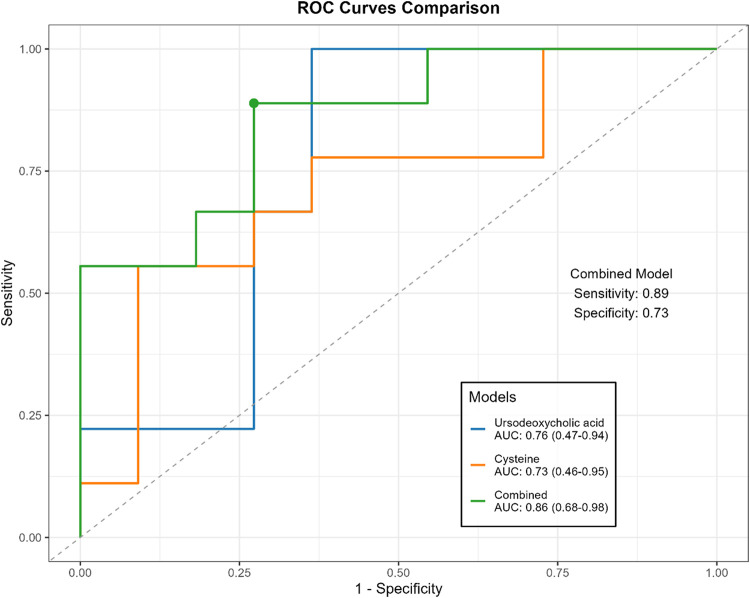
Comparison of ROC curves for ursodeoxycholic acid, cysteine and combined models.

The predictive ability of efficacy-related metabolites was evaluated using receiver operating characteristic (ROC) curves. Treatment response was classified into responders (pCR and pPR) and non-responders (pSD). Among these metabolites, ursodeoxycholic acid (AUC = 0.76 [0.53–0.95]) showed the highest predictive value, followed by cysteine (AUC = 0.73 [0.44–0.93]) (Table 6). When combined in a joint prediction model, the AUC increased to 0.86 (95% CI: 0.68–0.98), with a sensitivity of 0.89 and a specificity of 0.73 (Figure 7).

**TABLE 6:** Comparison of predictive performance indicators for efficacy-related metabolites.

Metabolite	AUC	95%CI	Sensitivity	Specificity
Ursodeoxycholic acid	0.76	0.47–0.94	1.00	0.64
Cysteine	0.73	0.46–0.95	0.56	0.91
Glycochenodeoxycholate	0.65	0.40–0.86	0.89	0.46
Adenosine	0.63	0.36–0.88	0.56	0.82
1-Oleoyl-sn-glycero-3-phosphocholine (LysoPC(18:1))	0.62	0.33–0.86	0.78	0.55
2-Keto-gluconic-acid	0.56	0.27–0.82	0.89	0.55
Epinephrine	0.56	0.27–0.83	0.56	0.82
Inosine-5-diphosphate	0.47	0.21–0.77	0.56	0.64
O-acetyl-serine	0.45	0.18–0.72	0.67	0.45
Combined^a^	0.86	0.68–0.98	0.89	0.73

Abbreviations: AUC, area under the curve; CI, confidence interval.

^a^ Combined: Combined predictive model of ursodeoxycholic acid and cysteine.

## 4 Discussion

Our metabolomics analysis revealed distinct temporal and efficacy-associated metabolic changes in HER2-breast cancer patients undergoing TEC NAC. To minimize inter-individual variability and more accurately capture treatment-induced alterations, we analyzed dynamic metabolite changes within each patient at three time points: pre-treatment (T1, before NAC), mid-treatment (T2, after 3 cycles of NAC), and post-treatment (T3, before surgery after 6 cycles of NAC). These changes reflect adaptive metabolic reprogramming in response to chemotherapy that could be related with tumor suppression.

Previous metabolomics studies have consistently indicated that amino acid and bile acid metabolism are closely associated with the efficacy of neoadjuvant chemotherapy (NAC) in breast cancer (Wei et al., 2013; Díaz et al., 2022; Yamada et al., 2024). Recent evidence has further validated these findings, showing that the biosynthesis pathway of leucine, valine, and isoleucine is significantly enriched in chemotherapy responders (Fang et al., 2025). Another study focusing on HER2-positive patients also revealed that taurine and bile acid–related metabolites were closely correlated with therapeutic efficacy (Zhang et al., 2024). Consistent with these reports, our study likewise observed significant alterations in leucine, valine, and multiple bile acid derivatives, suggesting that these metabolic features represent reproducible and consensus biomarkers of chemotherapy response.

During the early phase of NAC (T1 to T2), pathways related to nucleotide biosynthesis and tumor proliferation were significantly downregulated. Meanwhile, elevations in metabolites associated with vitamins and coenzymes may reflect shifts in cellular metabolic demands or stress adaptation. The later phase (T2 to T3) was characterized by the upregulation of energy and lipid metabolism, potentially supporting tumor cell survival under sustained therapeutic pressure.

Metabolism of branched-chain amino acids (BCAAs), glycerophosphocholine, and bile acids showed marked alterations during NAC treatment. Leucine and valine, two representative BCAAs, are essential nutrients for cancer growth, participating in biosynthetic processes and serving as energy substrates for tumor cells (DeBerardinis and Chandel, 2016). Elevated BCAAs concentrations have been reported in both plasma and tumor tissues of breast cancer patients, with increased expression of BCAAs catabolic enzymes in tumors compared to adjacent normal tissues, suggesting active utilization of these amino acids (Xu et al., 2023; Zhang and Han, 2017). In our study, leucine levels decreased while valine levels increased after NAC. Research shows that leucine promotes tumor proliferation by activating the mTOR signaling pathway and enhancing mitochondrial biogenesis and function (Chen et al., 2024). In contrast, elevated valine levels have shown a strong inverse association with breast cancer relapse, suggesting potential protective effects (Yang et al., 2024).

LysoPC(18:1) is a glycerophosphocholine (GPC), which has been found to be elevated in breast cancer tissues compared to benign tissues (Gribbestad et al., 1999). Elevated plasma lysophosphatidylcholine levels have been reported in murine models of metastatic breast cancer (Kus et al., 2018). Lysophosphatidylcholine binds to G protein-coupled receptors (G2A/GPR4), activating signaling pathways that promote cancer cell proliferation, migration, and survival, thereby enhancing tumor invasiveness and metastasis (Xu, 2002). Its conversion into other lysophospholipids further contributes to tumor progression (Xu, 2002). In our study, GPC levels showed a decreasing trend following NAC. Changes in LysoPC(18:1) were associated with tumor pathological response. LysoPC(18:1) levels showed the greatest decrease in pCR patients after three cycles of NAC, while only minimal changes were observed in the pSD group. Similarly, Cao et al. reported that patients showing a decline in GPC levels after NAC tended to have longer survival and were more likely to achieve partial tumor response (Cao et al., 2012). The reduction of pro-metastatic LysoPC(18:1) in pCR patients may signal attenuated tumor invasiveness, consistent with its role in driving metastatic pathways.

Bile acid metabolism also demonstrated strong associations with treatment efficacy. Breast cancer patients often exhibit elevated circulating bile acid levels compared to healthy individuals and those with benign breast conditions (Li et al., 2020; Anh et al., 2024). In our study, three bile acids—glycochenodeoxycholate, glycodeoxycholate, and ursodeoxycholate—were significantly reduced during NAC. Notably, pCR patients showed smaller reductions in glycochenodeoxycholate and increased levels of ursodeoxycholate, suggesting that preserved bile acid metabolism may be linked to better chemotherapy response. Previous studies also supported this finding, which reporting higher baseline levels of glycine-conjugated bile acids in NAC-sensitive TNBC patients and accumulation of glycochenodeoxycholate in Luminal A breast cancers with favorable prognosis (Díaz et al., 2022; Ta et al., 2019). Transcriptomic data showing that enhanced bile acid metabolism correlates with reduced proliferation and invasiveness, while its suppression is linked to more aggressive tumor phenotypes (Wu et al., 2022). These results suggest that greater decreases in bile acid levels during NAC may indicate poor therapeutic response.

Regarding the temporal trends observed in Supplementary Figure S1, the levels of LysoPC(18:1) and glycochenodeoxycholate markedly decreased from T1 to T2 and then stabilized or slightly increased from T2 to T3. This biphasic pattern may reflect two sequential processes. During the early phase of chemotherapy, acute metabolic disturbances in both tumor and host—such as rapid alterations in membrane phospholipid and bile acid metabolism—lead to the initial decline. As treatment continues, compensatory metabolic reprogramming, including hepatic functional recovery, restoration of lipoprotein metabolism, and reconstruction of the gut microbiota, may contribute to the subsequent stabilization or partial rebound of these metabolites. In our study, glycochenodeoxycholate levels in the pCR group showed a pronounced decrease from T1 to T2 followed by stabilization or mild rebound, whereas in the pPR and pSD groups, they continued to decline throughout the treatment period. This suggests distinct metabolic recovery trajectories across different response groups, with the pCR group exhibiting early metabolic perturbation followed by restoration of metabolic equilibrium, while persistent metabolic suppression in the pPR and pSD groups may indicate ongoing tumor burden and systemic stress.

Cysteine was most elevated in pSD patients during NAC. As a key precursor of glutathione, elevated cysteine may help neutralize chemotherapy-induced reactive oxygen species and enhance antioxidant capacity, thereby promoting tumor cell survival under treatment pressure (Bansal and Simon, 2018). *In vitro* studies have also shown that exogenous cysteine can stimulate breast cancer cell growth (Gu Y. et al., 2015). Furthermore, cysteine-derived hydrogen sulfide (H_2_S) supports cancer energy metabolism by modulating mitochondrial function (Serpa, 2020). These findings suggest that changes in cysteine levels during NAC may serve as a potential predictive biomarker for therapeutic response.

Ursodeoxycholic acid and cysteine demonstrated the highest predictive value in distinguishing responders from non-responders based on their changes from baseline after three cycles of NAC. A combined model incorporating both metabolites achieved an AUC of 0.86 (95%CI: 0.68–0.98), highlighting their potential utility as early indicators for treatment monitoring in breast cancer patients undergoing NAC.

Addtionally, neuroactive signaling molecules play the complex roles in breast cancer progression and treatment response. In our study, both acetylcholine (ACh) and epinephrine showed significant alterations during neoadjuvant chemotherapy (NAC). Plasma ACh levels were significantly decreased after three cycles of NAC (T2) and remained significantly lower at the end of six cycles (T3). ACh and its receptors have been implicated in tumor initiation and progression. In breast cancer, overexpression of ACh receptor subtypes such as α7-nAChR and α9-nAChR has been associated with enhanced proliferation, angiogenesis, and epithelial-mesenchymal transition (Lee et al., 2010; Chen et al., 2006). Tumor cells have been shown to autonomously synthesize ACh, establishing autocrine or paracrine loops that activate cholinergic receptors and promote malignancy (Wang et al., 2016; Song et al., 2003). The reduction in ACh levels may indicate that chemotherapy disrupts cholinergic signaling pathways involved in tumor progression. In contrast, epinephrine levels were significantly increased during the later phase of NAC (T2 to T3), with the most pronounced elevation observed in pCR patients and minimal changes in pSD patients. This observation appears to contrast with previous studies, which have reported that epinephrine promotes breast cancer progression and treatment resistance by activating adrenergic receptors and downstream signaling pathways (Zhou et al., 2020). This discrepancy may be attributed to the fact that plasma epinephrine levels do not fully reflect local adrenergic signaling activity within the tumor microenvironment. It is possible that elevated circulating epinephrine during chemotherapy represents a systemic stress response rather than a direct promoter of tumor progression. Further research is needed to elucidate the role of neurotransmitter in modulating chemotherapy response in breast cancer.

Taken together, our findings provide a comprehensive overview of the temporal metabolic reprogramming during NAC in HER2-negative breast cancer. The observed metabolic alterations may reflect adaptive mechanisms of tumor cells and systemic host responses under chemotherapeutic stress. In particular, the dynamic changes in cysteine and branched-chain amino acids suggest that redox balance and amino acid metabolism are closely associated with chemosensitivity, possibly through the regulation of glutathione synthesis and oxidative stress defense. Meanwhile, the involvement of bile acid metabolism implies potential crosstalk between hepatic detoxification and tumor metabolic adaptation. These findings collectively indicate that redox homeostasis and metabolic plasticity play central roles in determining treatment response. Mechanistic or functional validation was not performed in the present study. Future work will include extended clinical cohorts and *in vitro* models to further verify these findings and elucidate the causal roles of the identified metabolites and pathways in mediating NAC efficacy.

Despite offering valuable insights, our study has certain limitations. The limited sample size precluded stratified analyses based on molecular subtypes. Future research should validate these findings in larger cohorts and integrate multi-omics approaches to strengthen their clinical utility for personalized treatment strategies.

In conclusion, we conducted a longitudinal metabolomics study using UHPLC-HRMS in HER2-breast cancer patients receiving the TEC NAC regimen. Our findings revealed key metabolic pathways and specific plasma metabolites associated with NAC response. Branched-chain amino acid metabolism, choline-related substance metabolism, and bile acid metabolism were significantly altered during NAC. Notably, we identified response-associated metabolic signatures, with specific plasma metabolites demonstrating differential patterns across pCR, pPR, and pSD groups. Particularly, a predictive model incorporating ursodeoxycholic acid and cysteine changes from baseline to mid-treatment (T1-T2) demonstrated promising potential for distinguishing responders from non-responders. These findings provide a potential non-invasive approach for predicting NAC sensitivity and identifying patients most likely to benefit from this regimen.

## Data Availability

The original contributions presented in the study are included in the article/Supplementary Material, further inquiries can be directed to the corresponding authors.
