# Supplementary material for: Metabolomic changes associated with treatment response of neoadjuvant chemotherapy with TEC regimen in HER2-negative breast cancer
Source: Front Pharmacol. 2025 Nov 19;16:1707223. doi: 10.3389/fphar.2025.1707223 (PMC12673219; doi:10.3389/fphar.2025.1707223)

Supplementary Figure 1 Dynamic changes of metabolites in different efficacy groups. Lines demonstrate the changes in relative expression intensity of efficacy-related metabolites at three time points in three efficacy groups of patients. The horizontal coordinate is the treatment timepoint, the vertical coordinate is the expression intensity (relative abundance) of the normalized metabolites, and the connecting line between the time points reflects the continuous trend of metabolite changes. Response category is differentiated by color. pCR: pathological complete remission; pPR: partial remission; pSD: stable disease; T1: baseline; T2: after 3 cycles; T3: after 6 cycles

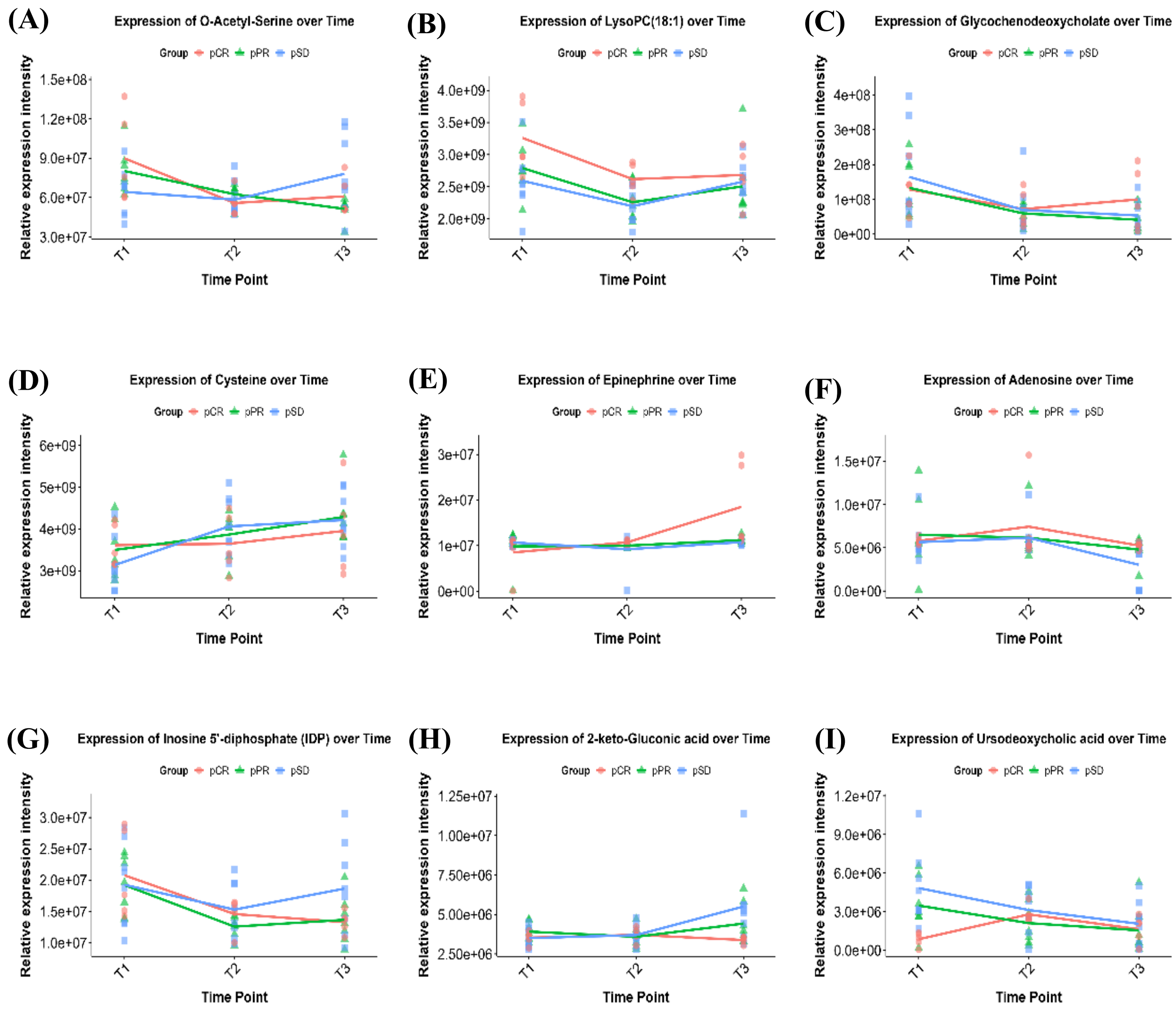

Supplement: Supplementary file 2 [file Image1.pdf]
